# The effect of transcranial direct current stimulation on contrast sensitivity and visual evoked potential amplitude in adults with amblyopia

**DOI:** 10.1038/srep19280

**Published:** 2016-01-14

**Authors:** Zhaofeng Ding, Jinrong Li, Daniel P. Spiegel, Zidong Chen, Lily Chan, Guangwei Luo, Junpeng Yuan, Daming Deng, Minbin Yu, Benjamin Thompson

**Affiliations:** 1 State Key Laboratory of Ophthalmology, Zhongshan Ophthalmic Center, Sun Yat-sen University, Guangzhou, China; 2 McGill Vision Research, McGill University, Canada; 3 School of Optometry and Vision Science, University of Auckland, New Zealand; 4 School of Optometry, The Hong Kong Polytechnic University, Hong Kong, SAR, China; 5 School of Optometry and Vision Science, University of Waterloo, Canada

## Abstract

Amblyopia is a neurodevelopmental disorder of vision that occurs when the visual cortex receives decorrelated inputs from the two eyes during an early critical period of development. Amblyopic eyes are subject to suppression from the fellow eye, generate weaker visual evoked potentials (VEPs) than fellow eyes and have multiple visual deficits including impairments in visual acuity and contrast sensitivity. Primate models and human psychophysics indicate that stronger suppression is associated with greater deficits in amblyopic eye contrast sensitivity and visual acuity. We tested whether transcranial direct current stimulation (tDCS) of the visual cortex would modulate VEP amplitude and contrast sensitivity in adults with amblyopia. tDCS can transiently alter cortical excitability and may influence suppressive neural interactions. Twenty-one patients with amblyopia and twenty-seven controls completed separate sessions of anodal (a-), cathodal (c-) and sham (s-) visual cortex tDCS. A-tDCS transiently and significantly increased VEP amplitudes for amblyopic, fellow and control eyes and contrast sensitivity for amblyopic and control eyes. C-tDCS decreased VEP amplitude and contrast sensitivity and s-tDCS had no effect. These results suggest that tDCS can modulate visual cortex responses to information from adult amblyopic eyes and provide a foundation for future clinical studies of tDCS in adults with amblyopia.

Abnormal binocular visual experience during early childhood can result in amblyopia, a neurodevelopmental disorder of the visual cortex^1^,^2^. Amblyopia impairs a wide range of visual functions including contrast sensitivity^3^, visual acuity^2^, stereopsis^4^,^5^, global motion perception^6^,^7^ and contour integration^8^ (see^9^,^10^ for reviews). Amblyopic eyes also exhibit high levels of crowding^11^,^12^ and abnormal, suppressive lateral spatial interactions that are related to the loss of visual acuity in the affected eye^13^,^14^,^15^. The neural basis of amblyopia is yet to be fully elucidated, however primate models of strabismic and anisometropic amblyopia have reported weaker responses and losses of spatial resolution within V1 when cells were driven by the amblyopic eye compared to the fellow eye^16^,^17^. However, these V1 deficits were not sufficient to explain the behavioral deficits in contrast sensitivity within the same animals and therefore it is likely that extrastriate areas are also affected^16^. Indeed, abnormal responses to inputs from the amblyopic eye have recently been reported in V2^17^,^18^,^19^ and MT^20^ in primate models of strabismic and anisometropic amblyopia. Strong interocular suppression has also been observed within V1 and V2 in primate models of strabismic^18^ and anisometropic^19^ amblyopia. Importantly, in these studies, the magnitude of suppression was closely related to the behavioral loss of contrast sensitivity in the amblyopic eye suggesting that interocular suppression may play an important role in the monocular loss of contrast sensitivity that occurs in primate models of amblyopia^18^,^19^.

The results of investigations into the neural basis of amblyopia in humans are broadly consistent with the primate neurophysiology data. Visual evoked potential (VEP) measurements have shown that stimulation of the amblyopic eye evokes a weaker cortical response than stimulation of the fellow eye^21^. Studies utilizing functional magnetic imaging (fMRI) have extended these findings to reveal that attenuated and abnormal responses to the amblyopic eye occur in V1^22^,^23^, the lateral geniculate nucleus^24^,^25^,^26^, and a range of extrastriate visual areas including V2^22^,^23^ and MT^27^,^28^. In addition, psychophysical evidence indicates that stronger suppression of the amblyopic eye by the fellow eye is correlated with poorer amblyopic eye visual acuity in human patients^29^,^30^,^31^,^32^. Current evidence also suggests that dichoptic treatment interventions designed to promote binocular vision and reduce suppression can improve aspects of both binocular (stereopsis) and monocular (visual acuity and contrast sensitivity) vision in adult patients with amblyopia^33^,^34^,^35^,^36^,^37^ (see^38^ for a recent review). Reduced visual cortex excitability measured using phosphene thresholds for single pulses of transcranial magnetic stimulation (TMS) has also been observed in patients with amblyopia^38^,^39^, possibly reflecting abnormally high levels of cortical inhibition. Together, these findings are consistent with the idea that suppression is a key component of the amblyopia syndrome.

Transcranial direct current stimulation (tDCS) is a non-invasive brain stimulation technique that can transiently alter the excitability of targeted brain areas in a polarity specific manner^40^. When applied to the motor cortex, anodal tDCS (a-tDCS) tends to increase cortical excitability, measured as an increase in the amplitude of motor evoked potentials elicited by single pulse TMS^40^. Cathodal tDCS (c-tDCS), on the other hand, tends to reduce motor cortex excitability^40^. Comparable polarity-specific effects of tDCS on excitability have been reported for the visual cortex^41^. For example, a-tDCS of the occipital poles transiently decreases TMS phosphene thresholds whereas c-tDCS has the opposite effect^42^,^43^.

One potential mechanism for the increase in cortical excitability following a-tDCS is a reduction in GABA-mediated inhibition within the stimulated region. Stagg *et al*.^44^ used magnetic resonance spectroscopy to assess the relative concentration of glutamate and GABA within the motor cortex before and after tDCS. A-tDCS reduced the concentration of GABA relative to glutamate whereas c-tDCS induced comparable reductions in both GABA and glutamate. The selective reduction of GABA concentration following motor cortex a-tDCS has subsequently been replicated^45^,^46^. GABA mediated inhibition has been linked to a number of suppressive neural interactions within the visual cortex such as those underlying surround suppression in humans and primates^47^,^48^,^49^, bistable perception in humans^50^ and interocular suppression in cats with experimentally induced strabismus^51^. Furthermore, GABA mediated inhibition has been identified as one of a number of mechanisms that regulate adult visual cortex plasticity in rodent models of deprivation amblyopia^52^. Together, these findings raise the possibility that a-tDCS may modulate visual cortex function in patients with amblyopia.

In an initial study, we found that a-tDCS of the occipital poles significantly reduced psychophysically measured surround suppression in adults with normal vision^53^. This result suggested that a-tDCS might act to transiently reduce GABA mediated inhibition within the visual cortex as had previously been reported for the motor cortex^44^. We subsequently observed that a-, but not c-tDCS, transiently improved amblyopic eye contrast sensitivity in 8 out of 13 adult patients with strabismic and/or anisometropic amblyopia^54^. A-tDCS had no effect on fellow eye contrast sensitivity. Functional MRI measurements in 5 of these patients indicated that a-, but not sham tDCS (s-tDCS) significantly reduced the response bias to inputs from the fellow eye vs. the amblyopic eye in V2 and V3^54^. In other words, a-tDCS reduced the cortical response asymmetry to inputs from the fellow and amblyopic eye in these patients.

Building on this previous work, the aim of this study was to further investigate the effects of tDCS on visual cortex responses and contrast sensitivity in a larger group of adults with amblyopia and controls. In particular, we addressed the following questions that were not part of our previous studies: 1) Are changes in amblyopic eye contrast sensitivity induced by tDCS associated with changes in the cortical response to inputs from the amblyopic eye? Cortical responses were measured using monocular pattern reversal VEPs. 2) Do the effects of tDCS on contrast sensitivity and VEP amplitude differ between patients with amblyopia and controls? 3) Do the effects of tDCS on contrast sensitivity and VEP amplitude differ significantly from a sham stimulation control condition?

## Materials and Methods

### Participants

Twenty-one adult participants with unilateral amblyopia (Table 1) and twenty-seven adults with normal vision (mean age 23.0 years, SD 2.3, range 19–30 years; 21 females) participated in this study. The Zhongshan Ophthalmic Center ethics committee approved the study and all study protocols were in accordance with the Declaration of Helsinki. Informed consent was obtained from all participants prior to data collection. Participants with amblyopia had an intraocular acuity difference of at least 0.2 LogMAR with no organic cause and 0.1 LogMAR visual acuity or better in the fellow eye. Participants with amblyopia were classified as having strabismic, anisometropic or mixed mechanism amblyopia (both strabismus and anisometropia). Anisometropia was defined as a spherical equivalent difference of 1 dioptre or more between the two eyes. Participants with normal vision had 0.1 LogMAR acuity or better in each eye and no history of visual disorders. Best refractive correction was worn during testing for all experimental sessions. No participants had a history of neurological or psychiatric disorder, any implanted medical devices or were currently taking medication.

Each participant took part in 7 experimental sessions; three VEP sessions, one each for anodal tDCS (a-tDCS), cathodal tDCS (c-tDCS) and sham tDCS (s-tDCS), and four contrast sensitivity sessions (familiarization, a-, c-, and s-tDCS). For control participants only one eye, selected at random, was tested.

### Transcranial Direct Current Stimulation

tDCS was delivered by a battery-driven constant-current stimulator (Chattanooga Ionto, USA) using a pair of conductive rubber electrodes (4 mm × 6 mm stimulating electrode, 5 mm × 7 mm reference electrode) housed in saline-soaked synthetic sponges. A-tDCS, c-tDCS or s-tDCS was applied with the stimulating electrode placed over Oz and the reference over Cz. Electrode size and placement was adopted from previous studies of visual cortex stimulation^53^,^54^. All participants with amblyopia and 12 control participants received 20 minutes of tDCS. A separate group of 15 control participants received 10 minutes of tDCS to provide initial dose-response data for visual cortex tDCS. A- and c-tDCS were delivered at 2 mA. Sham stimulation involved a 30 second ramp up of anodal stimulation after which the stimulator was shut off out of sight of the participant. Stimulation sessions were separated by at least 48 hours and a-, c- and s- tDCS were delivered in a random sequence. For the VEP experiment, VEPs were recorded immediately before (baseline), after and 30 minutes after stimulation. For the contrast sensitivity experiment, measurements were made immediately before (baseline), during, after and 30 minutes after stimulation.

### Contrast sensitivity measurements

Contrast sensitivity was measured using a 10 cpd Gabor patch (radius 1.3°, sigma 1°), presented on a uniform grey background (50 cd/m^2^) for 500 ms within a Gaussian temporal envelope (100 ms ramp up and 100 ms ramp down). A relatively high spatial frequency was chosen, as the effects of amblyopia on contrast sensitivity are most pronounced at high spatial frequencies^3^. On each trial, participants judged the orientation of the patch (vertical vs. horizontal). Stimuli were generated using Psykinematix software, which allows for 10.8 bits of contrast resolution, and presented on an Eizo CRT monitor (1024 × 768 resolution, 120 Hz refresh rate). The viewing distance was 200 cm and a tight fitting opaque patch was worn over the non-viewing eye.

A two-alternative forced choice (2AFC) paradigm and a 2-down-1-up adaptive staircase procedure (proportional step size of 25% before the first reversal and 7.5% increments and 15% decrements after the first reversal) was used to measure detection thresholds. Thresholds were calculated as the mean of the last four reversals out of a total of six reversals. Participants completed at least 1 hour of task familiarization in a separate session prior to the tDCS sessions. The order of the a-, c- and s-tDCS sessions was randomized across participants. Contrast sensitivity was measured before, during, after and 30 minutes after tDCS. Only a single spatial frequency was measured because tDCS can only be administered for a finite period of time and the after-effects can decay rapidly. Therefore we did not anticipate that there would be time to measure a full contrast sensitivity function.

### Pattern visual evoked potentials

The VEP stimuli were standard black (5 cd/m^2^) and white (80 cd/m^2^) pattern-reversal checkerboards generated with a Roland Consult clinical electrophysiology system viewed from 100 cm with a temporal frequency of 2 Hz. The stimuli were presented with a resolution of 1280 × 1024 at two contrasts (100% and 50% contrast) and two check sizes (15′ and 60′, equating to a fundamental spatial frequency of 2 cpd and 0.5 cpd respectively). VEPs for each of the 4 stimuli were collected at each time point (pre, post and 30 minutes post tDCS) in a random sequence. For participants with amblyopia, the fellow eye was always tested first. Measurements conformed to the ISCEV standards for clinical VEP recording. The non-viewing eye was occluded with a tight fitting opaque patch.

### Data analysis

Contrast detection thresholds were converted to log contrast sensitivity. VEP amplitude was defined as the difference between the N75 negative peak and the P100 positive peak amplitudes in μV. Using this difference rather than absolute values facilitated the comparison of VEP data across separate sessions. The N75 peak was defined as a negative peak 60 to 110 ms after the pattern reversal. The peak of the first positive wave after the N75 peak was defined as the P100. Latencies for both the N75 and P100 were also calculated. Changes from pre-stimulation baseline within each session were calculated by subtraction of the baseline value from the subsequent values within the session.

Four ANOVAs were conducted on the change from baseline data for contrast sensitivity to compare the effects of a-, c- and s-tDCS on the amblyopic, fellow fixing and control eyes and to compare the effects of 10 and 20 minutes of tDCS in control participants. 1) An ANOVA with factors of Eye (amblyopic vs. fellow fixing eye), Stimulation (anodal vs. cathodal vs. sham) and Time (during vs. post vs. post 30 minutes) was conducted on the results from the observers with amblyopia. 2) A mixed ANOVA with within-subject factors of Stimulation (anodal vs. cathodal vs. sham) and Time (during vs. post vs. post 30 minutes) and a between-subjects factor of Eye (amblyopic vs. control) was conducted on the data from participants with amblyopia and controls who received 20 minutes of tDCS. 3) A mixed ANOVA with within-subject factors of Stimulation (anodal vs. cathodal vs. sham) and Time (during vs. post vs. post 30 minutes) and a between-subjects factor of Eye (fellow fixing eye vs. control) was conducted on the data from participants with amblyopia and controls who received 20 minutes of tDCS. 4) A mixed ANOVA with within-subject factors of Stimulation (anodal vs. cathodal vs. sham) and Time (during vs. post vs. post 30 minutes) and a between-subjects factor of tDCS Duration (20 minutes vs. 10 minutes) was conducted on the data from controls who received either 20 or 10 minutes of tDCS.

Post-hoc paired samples t-tests were used to compare contrast sensitivity changes induced by c- or a-tDCS to the within session baseline and s-tDCS. Pearson’s correlation was employed to evaluate the relationship between the mean contrast sensitivity change (the mean of the during, post and post 30 minutes changes) and the severity of amblyopia.

The same analysis approach was applied to the VEP amplitude and latency data. Additional within-subjects factors of Spatial frequency (15′ vs 60′) and Contrast (50% vs. 100%) were added to the four ANOVAs and the ‘during stimulation’ timepoint was removed from the analysis as this was not collected for the VEP experiments due to technical constraints. Pearson’s correlation was employed to evaluate the relationship between the mean VEP change (the mean of post and post 30 minutes changes) and severity of amblyopia.

Finally Pearson’s correlations between the average changes in contrast sensitivity and the average changes in the VEP amplitude following anodal tDCS were conducted for four data sets; amblyopic eyes, fellow fixing eyes, control eyes (20 min tDCS) and control eyes (10 min tDCS).

## Results

We first assessed the effect of a-tDCS on pattern reversal VEPs (statistical results are provided in Tables 2 and 3). VEP amplitude was significantly weaker at baseline for amblyopic eyes than fellow eyes for each session. The mean baseline VEP amplitude for amblyopic eyes collapsed across stimulus contrast, spatial frequency and session was 6.95 ± 3.44 uV compared to 10.84 ± 5.09 uV for fellow eyes (t_20_ = 4.316, p < 0.001).

**Figure 1:**
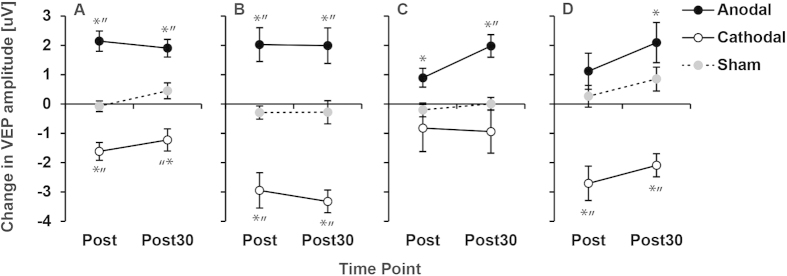
A-tDCS increased the amplitude of the pattern reversal VEP. The change in VEP amplitude from baseline after 20 minutes of anodal, cathodal or sham tDCS is shown for amblyopic (**A**; n = 21), non-amblyopic (**B**; n = 21) and control (**C**; n = 12) eyes. Positive values indicate an improvement. Measurements were made directly after (Post) and 30 minutes after (Post30) simulation. For controls, reducing the simulation duration to 10 minutes did not change the pattern of tDCS effects (**D**; n = 15). * = significant change from baseline, “ = significant difference from sham (paired t-test, p < 0.05).

**Figure 2:**
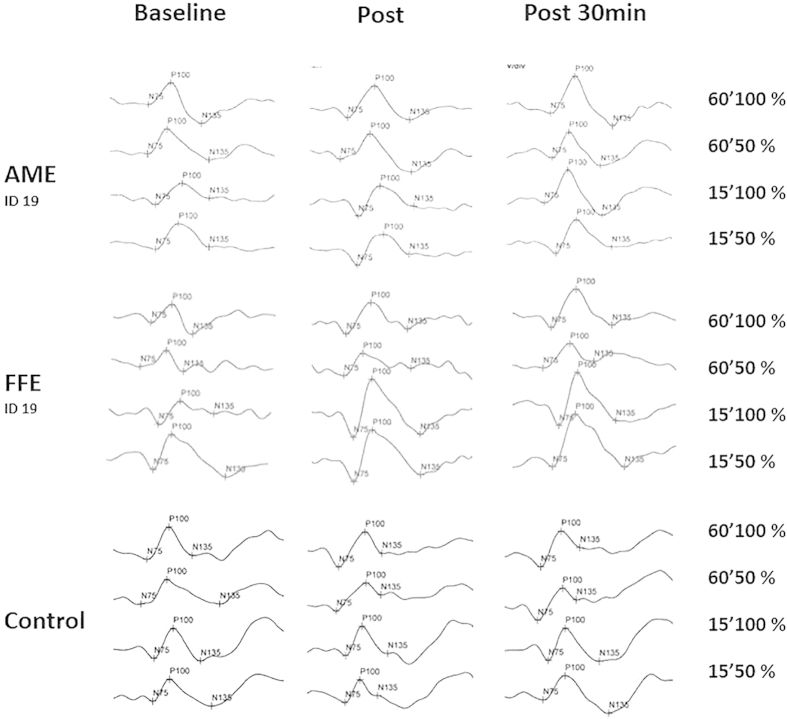
Example VEP Reponses for an amblyopic eye (Patient 19), a fellow eye (Patient 19) and a control eye before (baseline), after and 30 minutes after a-tDCS. The right column identifies the size of the check in the VEP stimulus (either 60′ or 15′) and the contrast (either 50% or 100%). Each waveform is the average of 64 repetitions.

A-tDCS increased the amplitude of the pattern reversal VEP for amblyopic, non-amblyopic and control eyes directly after and 30 minutes after stimulation (Fig. 1). This effect was significantly different from s-tDCS and c-tDCS and was not dependent on the spatial frequency or contrast of the standard checkerboard VEP stimulus. Conversely, c-tDCS reduced the amplitude of the VEP and sham had no effect. VEP latencies were unaffected by tDCS suggesting that the effect was restricted to the response of cortical neurons and did not influence conduction time from retina to cortex. For controls, reducing the stimulation duration to ten minutes did not significantly alter the effects, although the c-tDCS induced decreases in VEP amplitude were more pronounced. Representative VEP waveforms are shown in Fig. 2.

**Figure 3:**
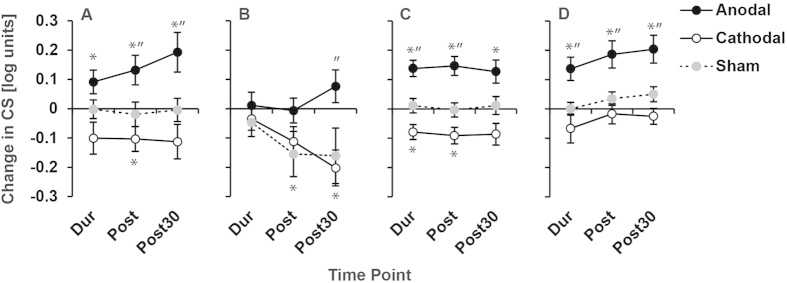
A-tDCS enhanced contrast sensitivity. The change in log contrast sensitivity relative to baseline after 20 minutes of anodal, cathodal or sham tDCS is shown for amblyopic (**A**), non-amblyopic (**B**) and control (**C**) eyes. Positive values indicate an improvement. Measurements were made during (Dur), directly after (Post) and 30 minutes after (Post30) simulation. For controls, reducing the stimulation duration to 10 minutes did not change the pattern of results (**D**). * = Significant change from baseline, “ = significant difference from sham (paired t-test, p < 0.05).

The results for contrast sensitivity were consistent with the VEP measurements. Amblyopic eye contrast sensitivity was significantly lower than that of the fellow eye for all baseline sessions (mean log sensitivity collapsed across sessions; amblyopic eye = 0.70 ± 0.52, fellow eye = 1.66 ± 0.34; t_20_ = 7.34, p < 0.001). A-tDCS significantly improved contrast sensitivity whereas c-tDCS had the opposite effect (Fig. 3; Tables 4 and 5). Contrast sensitivity measurements for two participants showed a large reduction in sensitivity following sham stimulation for the non-amblyopic eye condition only. This effect did not occur for other participants and is reflected in the standard error of the sham data in Fig. 3B.

**Figure 4:**
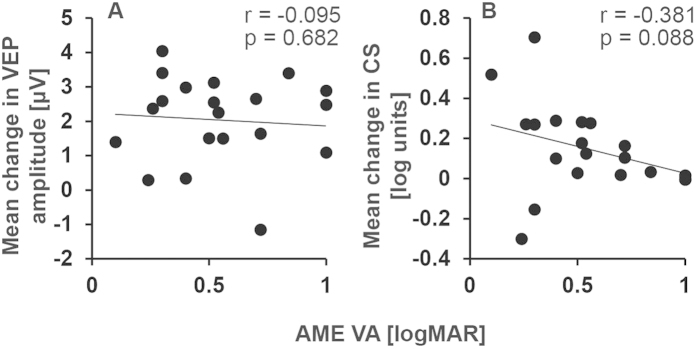
Relationships between amblyopia severity and tDCS induced changes in VEP amplitude (**A**) and contrast sensitivity (**B**).

**Figure 5:**
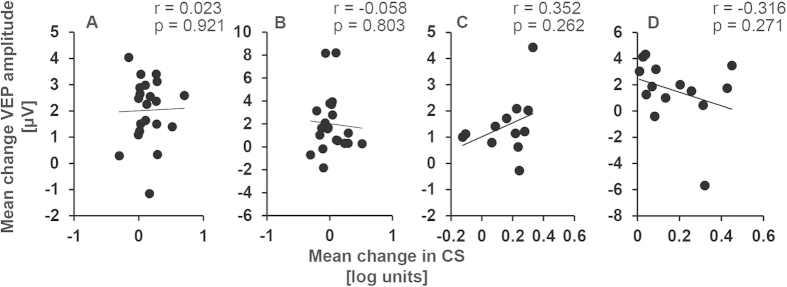
Correlations between the effects of anodal tDCS on contrast sensitivity and VEP amplitude for amblyopic eyes (Panel A), fellow fixing eyes (Panel B), control eyes 10 min tDCS (Panel C) and control eyes 20 minutes tDCS (Panel D). The change in contrast sensitivity and VEP amplitude were collapsed across time points.

No significant relationships were found between amblyopic eye visual acuity at baseline and the a-tDCS induced changes in contrast sensitivity (Fig. 4A) or VEP amplitude (Fig. 4B) for amblyopic eyes. Data were collapsed across the ‘during’, ‘post’ and ‘post 30’ time points for correlation analyses. Changes in contrast sensitivity induced by a-tDCS and the changes in VEP amplitude induced by a-tDCS were also not significantly correlated (Fig. 5).

Given the significant increases in VEP amplitude and contrast sensitivity following a-tDCS, we investigated the duration of the effects. For the VEP measurements, fourteen participants with amblyopia completed a-tDCS as their first or second tDCS session and therefore completed a baseline measurement 48 hours later. There was a significant increase in VEP amplitude from the a-tDCS baseline to the baseline 48 hours later (t_13_ = 3.8, p = 0.002) suggesting an enduring effect of the a-tDCS. This did not occur for sham stimulation (n = 12, t_11_ = 1.5, p = 0.2). Post a-tDCS baselines did not differ significantly from pre a-tDCS baselines for amblyopic eye contrast sensitivity (n = 13, t_12_ = 2.0, p = 0.07).

## Discussion

Our results demonstrate that a single session of a-tDCS can transiently increase VEP amplitude and contrast sensitivity in adult patients with amblyopia. Specifically, a-tDCS increased VEP amplitude and contrast sensitivity for amblyopic eyes. Similar effects were found for control eyes, however fellow eyes of patients with amblyopia did not show increased contrast sensitivity following a-tDCS. Importantly, increases did not occur following c- or s-tDCS, indicating that the effects were specific to a-tDCS and were not due to within session learning.

The results of this study contribute to a growing literature indicating that non-invasive brain stimulation can modulate contrast sensitivity in adults with amblyopia^39^,^54^,^55^. A possible mechanism for the a-tDCS effects we report is reduced inhibition within the visual cortex. Visual input from the amblyopic eye is subject to attenuation^56^ and suppression^18^,^19^,^31^ which may contribute to the weakened cortical response to information from the amblyopic eye^22^. The magnitude of suppression is positively correlated with the loss of visual acuity^29^,^30^,^31^,^32^ and contrast sensitivity^18^,^19^ in humans and primates with strabismic or anisometropic amblyopia, and reducing suppression may result in improvements in both visual acuity and stereopsis^33^,^36^,^57^. This suggests that suppression is an important contributor to the visual deficits associated with amblyopia. The inhibitory neurotransmitter GABA is involved in suppression of cortical inputs from one eye in cat models of strabismus^51^. GABA has also been implicated as one of a suite of mechanisms that gate recovery from amblyopia in adult rodents^52^. This role of GABA is directly relevant to a-tDCS effects because magnetic resonance spectroscopy measurements have shown that a-tDCS reduces the relative concentration of GABA when applied to the motor cortex^44^. Furthermore, a-tDCS induced reductions in motor cortex GABA have been associated with enhanced learning of a motor task, providing a link between changes in GABA and behavioral performance^46^. It is possible that a-tDCS reduces GABA within the visual cortex and therefore reduces chronic suppression of inputs from the amblyopic eye. This, combined with the excitatory effects of a-tDCS, may lead to a transient enhancement of the cortical response to amblyopic eye inputs in the form of an increased VEP amplitude and improved contrast sensitivity. Future work will test this hypothesis by assessing the effect of a-tDCS on measures of interocular suppression in patients with amblyopia.

We observed a dissociation between the a-tDCS induced increases in VEP amplitude that occurred for all eyes and the increases in contrast sensitivity that only occurred for amblyopic and control eyes. Fellow eyes did not show significant improvements in contrast sensitivity relative to baseline following a-tDCS. The increases in VEP amplitude are likely to reflect increased cortical excitability, a well-documented effect of a-tDCS within the motor cortex^40^. Therefore, our results suggest that the contrast sensitivity improvements induced by a-tDCS are not solely due to increased excitability. This finding raises the possibility that the improvements in contrast sensitivity may be more closely related to reduced inhibition/suppression within the visual cortex. While the control observers did not exhibit suppression under normal viewing conditions, a level of binocular competition or rivalry was introduced by the monocular viewing conditions used within the experiment (i.e. patching of the non-viewing eye). This may have been reduced by a-tDCS, leading to enhanced contrast sensitivity relative to baseline and sham. On the other hand, patching of the amblyopic eye would not induce rivalrous conditions for the fellow eye because suppressive interactions are biased in favor of the fellow eye^18^. Therefore no improvements in fellow eye contrast sensitivity would be expected following a-tDCS if reduced inhibition is involved.

An alternative explanation relates to the concept of homeostatic metaplasticity, whereby non-invasive brain stimulation acts to return a neural system to homeostasis^58^. Under this explanation, a-tDCS would have had a greater effect on neural populations preferring information from the amblyopic eye that have low levels of baseline activation. This explanation would also account for the trend towards more pronounced inhibitory effects of c-tDCS on fellow eye VEP amplitudes and contrast sensitivity. This is because fellow eye neural populations have a higher level of activation^22^ and are therefore likely to be more susceptible to the effects of inhibitory stimulation protocols. It is unclear how homeostatic metaplasticity relates to our control eye results. Based on the current data, we are not able to discriminate between these two explanations.

In addition to its effects on GABA, tDCS may also enhance expression of brain derived neurotropic factor (BDNF)^59^ and increased BDNF levels have been linked with recovery from adult amblyopia in animal models^60^. Furthermore, the aftereffects of tDCS rely on the function of NMDA receptors^61^. This has led to the suggestion that a-tDCS has long term potential (LTP) - like effects. NMDA receptor dependent changes in synaptic strength have been linked to recovery of ocular dominance plasticity animal models of amblyopia^62^ and may underlie the increases in VEP amplitude and contrast sensitivity we report. We favor an explanation based on temporary changes in inhibition and excitation because LTP and BDNF effects are gradual, whereas the effects we observed were rapid. It is possible, however, that LTP-like changes underlie the longer-term effects of a-tDCS on VEP amplitude that we observed for amblyopic eyes 48 hours after stimulation.

Previous studies into the effects of tDCS on the healthy visual cortex have generated conflicting results. In terms of VEPs, a-tDCS has been reported to increase and c-tDCS decrease VEP amplitude^63^, however opposite results have also been observed^64^. Furthermore, tDCS has been reported to have no effect on flash VEPs^65^. The results from previous studies investigating tDCS induced changes in contrast sensitivity are similarly variable. Antal *et al*.^66^ found that c-tDCS decreased static and dynamic contrast sensitivity, whereas a-tDCS had no reliable effect. Using threshold perimetry, Kraft *et al*.^67^ found that a-tDCS enhanced contrast sensitivity within the central 2 degrees of the visual field whereas c-tDCS had no effect. More recently Costa *et al*.^68^ also found that a-tDCS increased contrast sensitivity on threshold perimetry, but only in the periphery. Finally, an absence of acute tDCS effects on contrast sensitivity have been reported^53^,^69^. These discrepancies could be due to differences in visual stimuli, viewing conditions, tDCS parameters and electrode placement. However, individual differences between the participants taking part in each of these studies may also contribute to these discrepancies as a number of factors such as BDNF polymorphisms and hormone levels have been found to alter the response to tDCS^70^. In the current study, electrophysiological and behavioral measures were made in the same participants and the polarity specific effects were consistent across both measures; a-tDCS tended to increase VEP amplitude and contrast sensitivity whereas c-tDCS had the opposite effect. These results are in a good agreement with the established polarity dependent effects of motor cortex tDCS^40^ and support the use of tDCS to modulate visual cortex function.

Reducing the duration of tDCS to 10 minutes did not change the pattern of results in control participants. Whether this is also the case for patients with amblyopia remains to be tested. The effect of repeated doses of a-tDCS also remains an open question and the exploration of ways to prolong the effects of a-tDCS is an important next step in this field.

Although the effects of tDCS were consistent between the VEP and contrast sensitivity measurements, we did not find a direct correlation between the changes in VEP and changes in contrast sensitivity. This may be due to the very different nature of the stimuli used to elicit VEPs and measure contrast sensitivity. The lack of a direct correlation may also reflect different neural mechanisms for the two effects, as described above.

These results indicate that a-tDCS can temporarily increase contrast sensitivity in adults with amblyopia in agreement with previous studies^54^. It has also previously been reported that a-tDCS can enhance the effect of dichoptic treatment on stereopsis in adults with amblyopia^71^. Further work using standard clinical outcome measures such as visual acuity is required to assess whether a-tDCS alone has any clinical relevance for the treatment of amblyopia in adulthood.

## Additional Information

**How to cite this article**: Ding, Z. *et al*. The effect of transcranial direct current stimulation on contrast sensitivity and visual evoked potential amplitude in adults with amblyopia. *Sci. Rep.* **6**, 19280; doi: 10.1038/srep19280 (2016).

## Figures and Tables

**Table 1:** Clinical details of the participants with amblyopia.

ID	Current Age [Age of First Detection]/Gender	History of Previous Treatment	Type of Amblyopia	Visual Acuity [log MAR]	Current Refractive Error
01	17^15^/F	None	RE Aniso	0.30	+3.00 + 0.50 × 090
			LE	−0.18	+0.25 + 0.50 × 080
02	17^5^/M	None	RE Strab (26^Δ^ET)	0.24	+0.25 + 0.75 × 095
			LE	0.04	Plano + 1.00 × 080
03	17^8^/M	None	RE	−0.08	+0.25–0.50 × 175
			LE Mixed (9~17^Δ^ET)	0.70	+5.00 + 2.50 × 105
04	19^14^/M	None	RE	0.26	+6.25 + 0.50 × 090
			LE	0.08	+6.75 DS
05	17 [unknown]/ F	None	RE	−0.08	−0.25 DS
			LE Aniso	1.00	+5.00 + 1.50 × 115
06	21^5^/ M	Patching	RE Aniso	1.00	+5.25 + 0.75 × 025
			LE	0.00	+2.75 DS
07	17 [unknown]/F	None	RE	−0.08	+0.75 DS
			LE Aniso	0.40	+4.00 + 0.75 × 085
08	16^14^/F	None	RE Aniso	0.52	+4.50 + 1.75 × 070
			LE	0.00	−1.00 DS
09	16 [unknown]/M	None	RE Aniso	1.30	+6.25 DS
			LE	0.10	+0.25 DS
10	19^16^/M	None	RE Aniso	0.50	+1.75 + 2.50 × 090
			LE	−0.08	+1.00 + 2.00 × 088
11	18 [unknown]/ M	None	RE Aniso	0.30	+1.50–4.25 × 180
			LE	0.00	−1.50 DS
12	20 [unknown]/F	None	RE	0.00	+1.00 + 0.75 × 85
			LE Aniso	0.52	+3.25 + 0.75 × 60
13	22^19^/ F	None	RE	0.14	+1.00–1.75 × 015
			LE Aniso	0.40	+3.50–4.25 × 170
14	23^23^/F	None	RE	−0.1	+1.25 + 0.50 × 105
			LE Aniso	0.10	+4.50 + 1.75 × 115
15	25^6^/F	Patching	RE	−0.08	+4.25 DS
			LE Aniso	0.84	+5.75 + 0.50 × 105
16	21^6^/ M	Patching	RE	−0.08	+6.00 DS
			LE Strab (17^Δ^ET)	0.56	+5.75 + 0.25 × 160
17	17^7^/ F	Patching and Surgery	RE	0.00	Plano + 0.50 × 180
			LE Aniso	1.00	+6.50 + 1.75 × 105
18	19^19^/M	None	RE	−0.02	−3.25 DS
			LE Aniso	0.54	+4.50 + 1.50 × 100
19	23^7^/M	None	RE Aniso	0.30	+4.50 DS
			LE	0.00	Plano + 0.50 × 100
20	21^13^/M	Patching	RE	0.00	+0.25 + 0.25 × 090
			LE Aniso	0.72	+3.50 + 2.00 × 088
21	26^5^/F	None	RE	0.00	+5.00 DS
			LE Mixed (17^Δ^ET)	0.72	+6.00 DS

M, male, F, female, RE, right eye, LE, left eye, Aniso, anisometropic amblyopia, Strab, strabismic amblyopia, DS, diopter sphere.

**Table 2:** The results of ANOVAs testing the effect of tDCS on VEP amplitudes.

ANOVA factor(s)	AME vs. FEE	AME vs. Control	FFE vs. Control	Control 10 vs. 20 min
Stimulation (a-tDCS vs. c-tDCS vs. s-tDCS)	F_2, 40_ = 57.840, p < 0.001	F_2, 62_ = 36.227, p < 0.001	F_2, 62_ = 30.622, p < 0.001	F_2, 50_ = 21.441, p < 0.0001
Time (pre vs. post vs. 30 min post)	F_1, 20_ = 0.088, p = 0.769	F_1, 31_ = 4.462, p = 0.043	F_1, 31_ = 0.574, p = 0.454	F_1, 25_ = 17.490, p < 0.0001
Eye (amblyopic vs. fellow)	F_1, 20_ = 9.723, p = 0.005			
SF (low vs. high)	F_1, 20_ = 0.483, p = 0.495	F_1, 31_ = 2.415, p = 0.13	F_1, 31_ = 0.146, p = 0.705	F_1, 25_ = 0.005, p = 0.946
Contrast (low vs. high)	F_1, 20_ = 2.794, p = 0.609	F_1, 31_ = 2.093, p = 0.158	F_1, 31_ = 0.335, p = 0.567	F_1, 25_ = 11.092, p = 0.003
Stimulation*Time	F_2, 40_ = 0.804, p = 0.455	F_2, 62_ = 0.509, p = 604	F_2, 62_ = 1.643, p = 0.202	F_2, 50_ = 2.452, p = 0.98
Stimulation*Eye	F_2, 40_ = 3.985, p = 0.026			
Stimulation*SF	F_2, 40_ = 0.21, p = 0.811	F_2, 62_ = 0.672, p = 0.514	F_2, 62_ = 0.457, p = 0.635	F_2, 50_ = 0.176, p = 0.838
Stimulation*Contrast	F_2, 40_ = 0.765, p = 0.472	F_2, 62_ = 0.175, p = 0.84	F_2, 62_ = 0.531, p = 0.591	F_2, 50_ = 0.196, p = 0.818
Stimulation*Time*Eye	F_2, 40_ = 0.804, p = 0.454			
Stimulation*Eye (between-subjects factor)		F_2, 62_ = 1.476, p = 0.236	F_2, 62_ = 4.708, p = 0.012	
Stimulation*tDCS Duration (between-subjects factor)				F_2, 50_ = 2.767, p = 0.073

AME, amblyopic eye, FFE, fellow fixing eye.

**Table 3:** The results of within subjects t-tests comparing the effects of tDCS on VEP amplitudes to within session baselines and the sham condition.

VEP data t-tests		Post	Post30
AME	Anodal	t_20_ = 6.256, p < 0.001	t_20_ = 6.351, p < 0.001
	Cathodal	t_20_ = −5.276, p < 0.001	t_20_ = −3.225, p = 0.004
	Sham	t_20_ = −0.44, p = 0.662	t_20_ = 1.724, p = 0.1
	Anodal vs. Sham	t_20_ = 6.129, p < 0.001	t_20_ = 4.293, p < 0.001
	Cathodal vs. Sham	t_20_ = −4.107, p = 0.001	t_20_ = −4.188, p < 0.001
FEE	Anodal	t_20_ = 3.517, p = 0.002	t_20_ = 3.267, p = 0.004
	Cathodal	t_20_ = −4.912, p < 0.001	t_20_ = −8.596, p < 0.001
	Sham	t_20_ = −1.359, p = 0.189	t_20_ = −0.751, p = 0.461
	Anodal vs. Sham	t_20_ = 3.483, p = 0.002	t_20_ = 3.148, p = 0.005
	Cathodal vs. Sham	t_20_ = −4.852, p < 0.001	t_20_ = −5.263, p < 0.001
Control 20 min	Anodal	t_11_ = 2.792, p = 0.018	t_11_ = 5.190, p < 0.001
	Cathodal	t_11_ = −1.020, p = 0.33	t_11_ = −1.27, p = 0.23
	Sham	t_11_ = −0.868, p = 0.404	t_11_ = 0.018, p = 0.986
	Anodal vs. Sham	t_11_ = 2.781, p = 0.018	t_11_ = 5.067, p < 0.001
	Cathodal vs. Sham	t_11_ = −0.795, p = 0.443	t_11_ = −1.344, p = 0.206
Control 10 min	Anodal	t_14_ = 1.864, p=0.082	t_14_ = 3.296, p = 0.005
	Cathodal	t_14_ = −4.598, p < 0.0001	t_14_ = −5.297, p < 0.0001
	Sham	t_14_ = 0.76, p = 0.486	t_14_ = 2.091, p = 0.055
	Anodal vs. Sham	t_14_ = 0.961, p = 0.353	t_14_ = 1.363, p = 0.194
	Cathodal vs. Sham	t_14_ = −4.032, p = 0.001	t_14_ = −5.228, p < 0.001

AME, amblyopic eye, FFE, fellow fixing eye. Unless indicated with “vs. Sham” the post hoc t-tests compare each condition to the within session baseline. Positive t values indicate an increase in VEP amplitude and negative values indicate a decrease.

**Table 4:** The results of ANVOAs testing the effect of tDCS on log contrast sensitivity.

ANOVA factor(s)	AME vs. FEE	AME vs. Control	FFE vs. Control	Control 10 vs. 20 min
Stimulation	F_2, 40_ = 12.364, p < 0.001	F_2, 72_ = 24.798, p < 0.001	F_2, 72_ = 11.069, p < 0.001	F_2, 58_ = 26.909, p < 0.0001
Time	F_2, 40_ = 0.997, p = 0.378	F_2, 72_ = 0.326, p = 0.723	F_2, 72_ = 2.067, p = 0.134	F_2, 58_ = 2.174, p = 0.128
Eye	F_1, 20_ = 4.381, p = 0.049			
Stimulation*Time	F_4, 80_ = 3.516, p = 0.011	F_4, 144_ = 0.473, p = 0.755	F_4, 144_ = 1.657, p = 0.163	F_2, 116_ = 0.156, p = 0.960
Stimulation*Eye	F_2, 40_ = 0.936, p = 0.4			
Stimulation*Time*Eye	F_4, 80_ = 0.321, p = 0.863			
Stimulation*Eye (between-subjects factor)		F_2, 72_ = 0.056, 0.946	F_2, 72_ = 0.796, p = 0.455	
Stimulation*tDCS Duration (between-subjects factor)				F_2, 58_ = 0.102, p = 0.903

Data reported as in Table 2.

**Table 5:** The results of t-tests comparing the effects of tDCS on log contrast sensitivity to the within session baseline and the sham condition.

VEP data t-tests		Dur	Post	Post30
AME	Anodal	t_20_ = 2.262, p = 0.035	t_20_ = 2.623, p = 0.016	t_20_ = 2.855, p = 0.01
	Cathodal	t_20_ = −1.841, p = 0.08	t_20_ = −2.453, p = 0.023	t_20_ = −1.903, p = 0.072
	Sham	t_20_ = −0.66, p = 0.948	t_20_ = −0.463, p = 0.648	t_20_ = −0.107, p = 0.916
	Anodal vs. Sham	t_20_ = 1.593, p = 0.127	t_20_ = 2.429, p = 0.025	t_20_ = 2.362, p = 0.028
	Cathodal vs. Sham	t_20_ = −1.375, p = 0.184	t_20_ = −1.729, p = 0.99	t_20_ = −1.451, p = 0.162
FEE	Anodal	t_20_ = 0.257, p = 0.8	t_20_ = −0.146, p = 0.885	t_20_ = 1.367, p = 0.187
	Cathodal	t_20_ = 0.892, p = 0.383	t_20_ = −2.225, p = 0.038	t_20_ = −3.294, p = 0.004
	Sham	t_20_ = −0.963, p = 0.347	t_20_ = −2.007, p = 0.058	t_20_ = −1.696, p = 0.105
	Anodal vs. Sham	t_20_ = 0.97, p = 0.343	t_20_ = 1.845, p = 0.08	t_20_ = 2.372, p = 0.028
	Cathodal vs. Sham	t_20_ = 0.229, p = 0.821	t_20_ = 0.411, p = 0.685	t_20_ = −0.382, p = 0.706
Control 20 min	Anodal	t_16_ = 4.945, p < 0.001	t_16_ = 4.550, p < 0.001	t_16_ = 3.208, p = 0.005
	Cathodal	t_16_ = −3.064, p = 0.007	t_16_ = −3.240, p = 0.005	t_16_ = −2.351, p = 0.032
	Sham	t_16_ = 0.437, p = 0.668	t_16_ = −0.149, p = 0.883	t_16_ = 0.369, p = 0.717
	Anodal vs. Sham	t_16_ = 4.095, p = 0.001	t_16_ = 3.244, p = 0.005	t_16_ 6 = 2.165, p = 0.046
	Cathodal vs. Sham	t_16_ = −2.456, p = 0.026	t_16_ = −2.358, p = 0.031	t_16_ = −1.866, p = 0.081
Control 10 min	Anodal	t_13_ = 3.449, t = 0.004	t_13_ = 4.007, p = 0.001	t_13_ = 4.282, p = 0.001
	Cathodal	t_13_ = −1.346, p = 0.201	t_13_ = −0.489, p = 0.633	t_13_ = −0.938, p = 0.365
	Sham	t_13_ = 0.017, p = 0.986	t_13_ = 1.539, p = 0.148	t_13_ = 1.961, p = 0.072
	Anodal vs. Sham	t_13_ = 2.874, p = 0.013	t_13_ = 2.833, p = 0.014	t_13_ = 2.860, p = 0.013
	Cathodal vs. Sham	t_13_ = −1.368, p = 0.194	t_13_ = −1.168, p = 0.264	t_13_ = −2.007, p = 0.066

Data reported as in Table 3.
